# Ultra-Pressurized Deposition of Hydrophobic Chitosan Surface Coating on Wood for Fungal Resistance

**DOI:** 10.3390/ijms252010899

**Published:** 2024-10-10

**Authors:** Suelen P. Facchi, Débora A. de Almeida, Karen K. B. Abrantes, Paula C. dos S. Rodrigues, Dauri J. Tessmann, Elton G. Bonafé, Marcelo F. da Silva, Mazeyar P. Gashti, Alessandro F. Martins, Lúcio Cardozo-Filho

**Affiliations:** 1 Graduate Program in Agronomy, State University of Maringá (UEM), Maringá 87020-900, Paraná, Brazil; spfacchil@gmail.com (S.P.F.); kkelibarbosa@gmail.com (K.K.B.A.); paulaesrodrigues@gmail.com (P.C.d.S.R.); djtessmann@uem.br (D.J.T.); lcfilho@uem.br (L.C.-F.); 2 Laboratory of Materials, Macromolecules, and Composites, Federal University of Technology-Paraná (UTFPR), Apucarana 86812-460, Paraná, Brazil; debora.a.almeida.93@gmail.com (D.A.d.A.); eltonbonafe@utfpr.edu.br (E.G.B.); marcelosilva@utfpr.edu.br (M.F.d.S.); 3 Department of Chemistry, State University of Maringá (UEM), Maringá 87020-900, Paraná, Brazil; 4 National Institute for Materials Advancement (NIMA), Pittsburg State University (PSU), Pittsburg, KS 66762, USA; 5 Department of Chemistry, Pittsburg State University (PSU), Pittsburg, KS 66762, USA

**Keywords:** biopolymers, thin films, hydrophobic coatings

## Abstract

Fungi (*Neolentinus lepideus*, *Nl*, and *Trametes versicolor*, *Tv*) impart wood rot, leading to economic and environmental issues. To overcome this issue, toxic chemicals are commonly employed for wood preservation, impacting the environment and human health. Surface coatings based on antimicrobial chitosan (CS) of high molar mass (145 × 10^5^ Da) were tested as wood preservation agents using an innovative strategy involving ultra-pressurizing CS solutions to deposit organic coatings on wood samples. Before coating deposition, the antifungal activity of CS in diluted acetic acid (AcOOH) solutions was evaluated against the rot fungi models *Neolentinus lepideus* (*Nl*) and *Trametes versicolor* (*Tv*). CS effectively inhibited fungal growth, particularly in solutions with concentrations equal to or higher than 0.125 mg/mL. Wood samples (*Eucalyptus* sp. and *Pinus* sp.) were then coated with CS under ultra-pressurization at 70 bar. The polymeric coating deposition on wood was confirmed through X-ray photoelectron spectroscopy (XPS), energy dispersive X-ray spectroscopy (EDS), scanning electron microscopy (SEM) images, and water contact angle measurements. Infrared spectroscopy (FTIR) spectra of the uncoated and coated samples suggested that CS does not penetrate the bulk of the wood samples due to its high molar mass but penetrates in the surface pores, leading to its impregnation in wood samples. Coated and uncoated wood samples were exposed to fungi (*Tv* and *Nl*) for 12 weeks. In vivo testing revealed that *Tv* and *Nl* fungi did not grow on wood samples coated with CS, whereas the fungi proliferated on uncoated samples. CS of high molar mass has film-forming properties, leading to a thin hydrophobic film on the wood surface (water contact angle of 118°). This effect is mainly attributed to the high molar mass of CS and the hydrogen bonding interactions established between CS chains and cellulose. This hydrophobic film prevents water interaction, resulting in a stable coating with insignificant leaching of CS after the stability test. The CS coating can offer a sustainable strategy to prevent wood degradation, overcoming the disadvantages of toxic chemicals often used as wood preservative agents.

## 1. Introduction

Wood is extensively used in construction primarily due to its sustainability and economic factors compared to other materials and its excellent thermal and insulation properties. Another positive factor that favors using wood in construction is that many species exhibit high resistance to the action of biodeterioration organisms. However, these species are insufficient to meet the growing demand for wood and derived materials. For this reason, wood species such as Pinus and Eucalyptus are extensively used due to their rapid growth from reforestation. Currently, about 70% of the wood consumed worldwide comes from reforestation. Therefore, the production of Pinus and Eucalyptus aims to meet the high market demand, especially in the civil construction field [1].

The issue is that Pinus and Eucalyptus species have low resistance to biodeteriorating agents, especially fungi, requiring pre-treatment before use [2]. Preservative treatments aim to increase the lifespan of the wood against fungi, insects, and other xylophagous organisms. However, the effective commercially used preservatives (e.g., azoles, quaternary ammonium salts, and boron salts, including sodium octaborate, sodium tetraborate, and sodium pentaborate) have disadvantages. Azoles and quaternary ammonium salts are economically costly. At the same time, boron-based compounds, which offer the most effective wood preservative systems currently available due to their broad spectrum of properties and effectiveness against bacteria, fungi, and insects, and their low toxicity in mammals, have water solubility and are easily leached from treated wood [1,3]. The leaching of these traditional wood preservatives from the wood decreases their effectiveness and can negatively impact the environment by accumulating in it [4].

Chemical agents must exhibit resistance to leaching (water insolubility) and low volatility and should not affect the properties of the wood (physical, chemical, mechanical, organoleptic, and decorative characteristics). They should not be corrosive to avoid damaging and compromising joints (straps, nails, etc.). They should reduce or not increase the flammability of the wood, as one of the disadvantages of wood is its high flammability. Additionally, they must be affordable, available in the market, and safe, presenting low cytotoxicity to humans, animals, and the environment [1,3,5]. Achieving all these properties is a significant challenge, even when synthetic chemical agents are employed.

Many studies have reported the use of bio-based materials, focusing on sustainability. Plant and fruit extracts have demonstrated significant potential as wood preservatives. Methanolic extracts from the leaves and bark of *Cleistanthus collinus* and *Prosopis juliflora* inhibit the growth of white rot and brown rot fungi, which are model organisms used in biodeterioration assays [6]. Other examples include extracts from mimosa bark (*Acacia mollissima*) and quebracho (*Schinopsis lorentzii*) [7], lichen [8], heartwood of teak (*Tectona grandis*) [9], propolis [10], and peels of fruits and vegetables [11]. Research on natural extracts has been extensive and has proven effective in resisting biodeterioration. However, like traditional boron-based preservation agents, these extracts are easily leached from the wood into the environment. This disadvantage remains a significant challenge to overcome. Another issue is that many of these materials are not commercially available on a large scale.

Extracts from the wood, such as tannins (TN), dyes, oils, resins, waxes, and fatty acids, isolated or combined with solvents and other additives, have also been used to preserve wood [12]. Tannins are well-recognized for their antifungal properties and have been tested, both in isolation and in combination with additives, as wood preservatives and alternatives to traditional agents. However, tannins are water-soluble and have difficulty adhering to the wood after treatment. Additives have been used to retain them in the treated wood [4,13,14,15].

On the other hand, few studies have reported using chitosan (CS) associated with additives as a wood preservative. CS is a commercial polysaccharide well recognized for its antifungal and antimicrobial activity against various bacteria [16,17,18,19]. It is a by-product of the crustacean processing industries, primarily shrimp, crab, and lobster. One study showed the impregnation of wood (*Pinus sylvestris* L.) with CS (degree of acetylation of 22%) of low (35 kDa, 67 kDa, and 70 kDa) and high molecular weights (215 kDa) to protect the wood against the fungi *Poria placenta* (brown rot), *Coniophora puteana* (brown rot), and *Coriolus versicolor* (white rot). The uptake of CS in the wood increased when low molecular weight CS was used, but the relative recovery of CS was lower due to its low molar mass in the wood after leaching. High molecular weight CS was more easily recovered after leaching in wood. For example, it can be precipitated in alkaline solutions. High molecular weight CS was more efficient against decay fungi than low molecular weight CS. The leaching of CS can be considered insignificant for high molecular weight samples, even when soaking the treated wood in aqueous media under vacuum (30 TORR). This is because high molecular weight CS is insoluble in water, presenting greater leaching resistance than low-molar mass CS. The fungi tested on CS-amended nutrient agar medium were inhibited at a 1% (*w*/*v*) concentration. In decay studies using small wood blocks, a 4.8% (*w*/*v*) CS concentration gave the best protection against brown rot fungi [16]. However, this study demonstrated wood impregnation with CS using a vacuum (30 Torr). Additionally, there is no physicochemical characterization of the CS-modified wood.

Other studies demonstrate using CS as a preservation agent, treating wood by simply impregnating the samples in CS solutions obtained in diluted acetic acid at ambient pressure. Polymers of high molar mass have low mobility in solution, especially CS solutions that present high viscosity even at low concentrations. Therefore, impregnating wood under vacuum or other low-pressure conditions is not ideal, as the wood is impregnated with CS weakly associated with its structure, potentially leading to leaching. This simple impregnation results in low protection effectiveness, as studies show contamination of the wood treated with CS under these conditions when the wood samples are incubated in contact with rot-causing fungi [20].

To overcome the problem of leaching of low-molar-mass CS (50–190 kDa), another study showed the application of CS crosslinked with genipin as a protective agent for wood against brown-rot fungi, *Gloeophyllum trabeum* and *Rhodonia placenta*, and two white-rot fungi, *Trametes versicolor* (*Tv*) and *Irpex lacteus* [21]. However, crosslinking further promotes leaching since it involves the formation of covalent bonds between amino groups of adjacent CS chains. Free amino groups in uncrosslinked CS are essential for stability because they interact with each other and with the wood’s cellulose and hemicellulose. This interaction leads to the stability of the impregnated CS through the deposition of a film on the wood, supported by intermolecular interactions, primarily hydrogen bonding. These intermolecular interactions are further enhanced as the molar mass increases.

Therefore, this study proposes using ultra-high molar mass CS as a wood preservative, employing the technique of ultra-pressurization to deposit thin films (surface coatings) on *Eucalyptus* wood. This novel strategy for applying organic coatings to solid substrates offers an alternative to the traditional layer-by-layer deposition method [22]. The ultra-pressurization strategy represents a significant innovation in developing surface coatings on wood samples. This method employs high mechanical pressures to facilitate the impregnation of CS on wood surfaces and pores [23]. The ultra-pressurization of wood samples in contact with CS solution can lead to the deposition of thin and uniform coatings, even using CS of high molecular weight. Additionally, ultra-pressurization enables the use of less toxic agents, aligning with sustainable practices that are increasingly valued across various industrial sectors. Ultra-pressurization is applicable to a wide range of civil construction materials, including plastics, ceramics, and metals [24].

The high-pressure condition may also facilitate the deposition of preservative agents, eliminating the need for thermal and high-vacuum treatments and avoiding the disadvantages of the traditional LbL method, which requires alternating immersion of solid substrates in solutions of oppositely charged polymers to form polyelectrolyte multilayers.

Additionally, this innovative approach differs from previous studies, which demonstrated the efficacy of CS in protecting wood but had disadvantages due to the use of low molar mass CS and vacuum or low-pressure conditions. We propose using ultra-high molar mass CS because of its insolubility in aqueous media and solubility in diluted acetic acid solutions. The ultra-pressurization method deposits CS on the surface of *Eucalyptus* sp. wood and impregnates it into the surface pores of the wood samples. The antifungal activity of CS is investigated against model fungi (*Neolentinus lepideus* (*Nl*) and *Tv*) used to demonstrate the fungicidal activity of wood preservation agents [25,26,27].

Wood samples (*Eucalyptus* sp. and *Pinus* sp.) are coated with ultra-high molar mass CS at 70 bar and characterized using water contact angle (WCA) measurements, energy dispersive X-ray spectroscopy (EDS), X-ray photoelectron spectroscopy (XPS), scanning electron microscopy (SEM), and infrared spectroscopy (FTIR) for the first time. The preservative potential of the coatings is evaluated through in vivo tests for 12 weeks, incubating the CS-coated wood samples in the presence of *Nl* (brown rot) and *Tv* (white rot) fungi. The results are compared with control assays using acetic acid (AcOOH) and tannin extracts as preservative agents for the tested wood samples. This study represents the first exploration of an ultra-pressurization method to develop organic coatings based on ultra-high molar mass CS, tested as wood preservatives. These coatings are crucial for enhancing moisture resistance and protecting wood against degradation agents, thereby increasing the material’s durability.

## 2. Results and Discussion

### 2.1. Characterization of the Surface Coatings Deposited on Wood

These WCA measurements were carried out on both uncoated and coated samples (Table 1) with the primary objective of assessing wettability. This assessment was conducted using the sessile drop method, where a water droplet was deposited on the wood surfaces, and three WCA measurements were obtained for each sample at different areas (Figure S1). The results indicate that the surfaces coated with CS exhibit hydrophobicity with WCAs higher than 90°. In contrast, the uncoated, TN-coated, and AcOOH-treated surfaces are hydrophilic, spreading water across their surfaces and resulting in WCAs below 90° (Table 1) [28].

WCAs are influenced by the coating type and the contact duration between the water droplet and the surface. The WCA measured on *Eucalyptus* sp. coated with CS is 118° after a 5 s contact with the water droplet (*p* ≤ 0.05). There is no significant change in WCAs on the CS-coated samples within 5 s. Despite its insolubility in water, CS-based materials can adsorb water molecules due to polar groups (-OH, -NH_2_, and -NH_3_^+^) in their structure [29,30].

However, CS deposition on the wood promotes hydrophobic surfaces. CS coatings decrease the interactions between the wood surfaces and water molecules, resulting in hydrophobic surfaces. Wood is composed of cellulose, hemicellulose, and tannins. The interaction between CS and cellulose/hemicellulose, the primary constituents of wood, significantly influences the wettability. Both cellulose and hemicellulose are polysaccharides with structures similar to CS. The intermolecular interactions, particularly hydrogen bonds, between the hydrophilic groups of these polysaccharides should reduce the availability of CS’s hydrophilic groups to water molecules. Consequently, this effect decreases the surface wettability of CS-coated samples, supporting hydrophobic surfaces and WCAs higher than 90° (Figure S1, Supplementary Materials). This observed result occurs for both *Pinus* sp. and *Eucalyptus* sp. samples.

WCA measurements on wood samples treated with AcOOH and coated with TN reveal values below 90°, indicating wettability. For *Eucalyptus* sp., WCA measurements after 5 s are 41° and 86° when treated with AcOOH and coated with TN, respectively (*p* ≤ 0.05). In the case of *Pinus* sp. treated with AcOOH, the water droplet is absorbed upon contact, and the WCA measured on TN-coated *Pinus* sp. is 61° after 5 s. The molecules of AcOOH and primarily hydrolyzable TN penetrate the internal wood fibers, enhancing interactions with water molecules and increasing wettability. This effect is more pronounced in *Pinus* sp. than in *Eucalyptus* sp. because of the lower density of *Pinus* sp. compared to *Eucalyptus* sp. AcOOH and TN significantly enhance surface wettability, resulting in WCAs below 90° [29,30].

The presence of AcOOH in *Pinus* sp. results in a super-hydrophilic surface, as AcOOH does not form a thin film that acts as a barrier to water absorption like CS. This explains the enhanced wettability observed in AcOOH-treated samples compared to those coated with CS and TN. Consequently, using the terms “wood treated” with AcOOH and “wood coated” with TN or CS is appropriate.

The alteration in wettability for both *Pinus* sp. and *Eucalyptus* sp., following polymer coating deposition, confirms the adsorption of these polymers onto the wood samples. WCA measurements provide evidence of CS and TN polymers on the wood surfaces, which should lead to thin films. These deposited films (coatings) can serve as effective barriers, preventing the deposition and growth of various xylophagous microorganisms in wood tissues and acting against biodeterioration.

**Figure 1:**
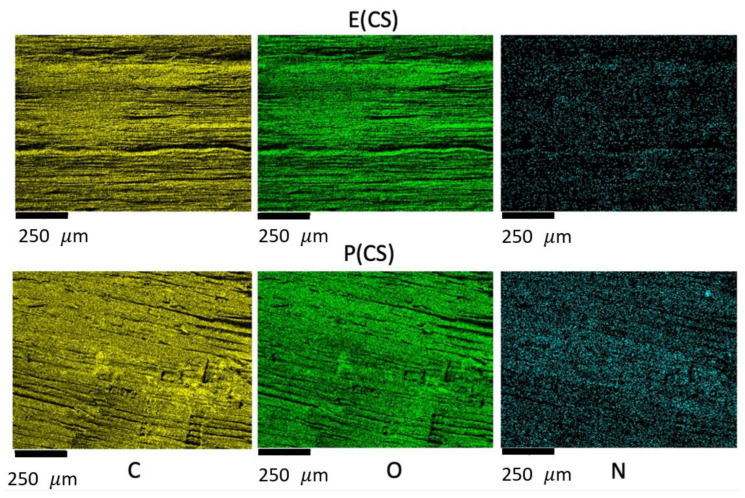
Elemental mapping of carbon (C), oxygen (O), and nitrogen (N) obtained on the CS-coated surfaces of *Eucalyptus* sp. (E(CS)) and *Pinus* sp. (P(CS)).

Elemental mapping of carbon, oxygen, and nitrogen atoms on the CS-coated wood surface confirms the presence of surface coatings (Figure 1). The nitrogen mapping indicates the deposition of CS in the samples, as nitrogen is present in the -NH_2_/-NH_3_^+^ groups of CS (Figure S2). EDS mapping further confirms a uniform distribution of nitrogen on the surfaces of both *Pinus* sp. and *Eucalyptus* sp. samples. This suggests that ultra-pressurization is an effective strategy for depositing surface coatings on wood samples.

**Figure 2:**
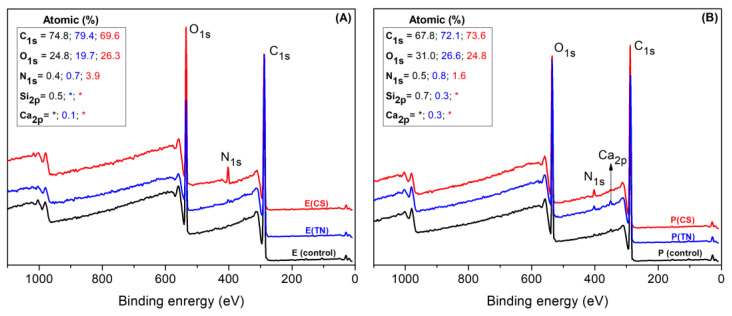
Survey XPS spectra performed on uncoated (P and E) and coated wood samples. *Eucalyptus* sp. coated with CS and TN are denoted as E(CS) and E(TN), respectively (**A**). *Pinus* sp. coated with CS and TN are labeled P(CS) and P(TN), respectively (**B**). Uncoated *Pinus* sp. and *Eucalyptus* sp. are represented as P and E, respectively. The asterisks (*) indicate that the elements Si and Ca were not detected by XPS.

**Figure 3:**
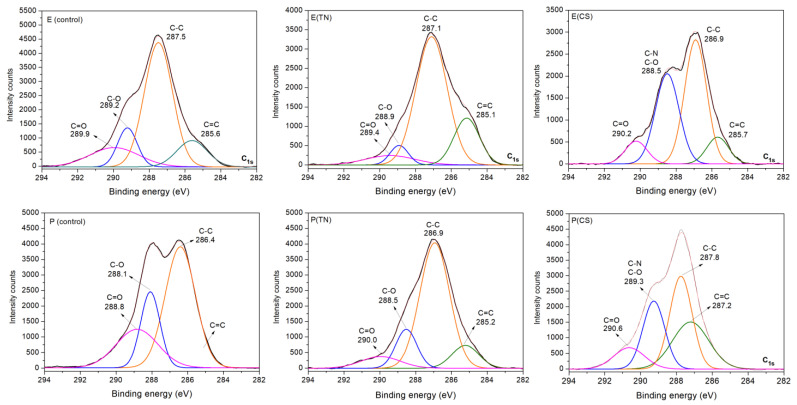
High-resolution XPS spectra of carbon (C_1s_) obtained on the uncoated (control) and coated wood samples. Sample labels include E: *Eucalyptus* sp.; P: *Pinus* sp.; CS: chitosan; TN: tannins; E(CS): *Eucalyptus* sp. coated with CS; E(TN): *Eucalyptus* sp. coated with TN; P(CS): *Pinus* sp. coated with CS; P(TN): *Pinus* sp. coated with TN.

Survey and high-resolution XPS spectra confirm the deposition of CS and TN onto *Eucalyptus* sp. and *Pinus* sp. (Figure 2 and Figure 3). The survey XPS spectra show peaks corresponding to nitrogen (N1s at 400 eV). The relative percentages of N_1s_ on the CS-coated surfaces are higher due to amine moieties on CS chains, indicating 3.9% and 1.6% upon *Eucalyptus* sp. and *Pinus* sp., respectively (Figure 2). TN-coated surfaces also exhibit N_1s_ peaks, as nitrogen is a macronutrient for plant development. The presence of CS and TN coatings is further confirmed by changes in the relative percentages of carbon (C_1s_ at 284 eV) and oxygen (O_1s_ at 531 eV) on the sample surfaces (Figure 2).

High-resolution XPS spectra of C_1s_ confirm the surface modifications of the wood samples. The spectrum profiles exhibit significant changes following polymer deposition. Both uncoated and coated samples display XPS spectra indicating chemical sites, such as C=C in phenolic compounds, C-C in saturated carbon chains, C-O in ether, and C=O in amide and carbonyl groups of hydrolyzable tannins. These chemical groups are characteristic of polysaccharides (cellulose, hemicellulose, and CS), lignin (phenolic compounds), and tannins (condensed and hydrolyzable).

While the spectral differences are less pronounced for uncoated and TN-coated samples, owing to the similar chemical composition of wood with the main components of TN (condensed and hydrolyzable tannins, Figure S3), CS deposition promotes peaks associated with C-O and C-N bonds. These bonds are indicative of CS’s chemical structure. Furthermore, the C=O peak prominently occurs in the C_1s_ spectra of CS-coated samples due to the degree of acetylation (15%) and C=O amide groups on CS (Figure S2).

**Figure 4:**
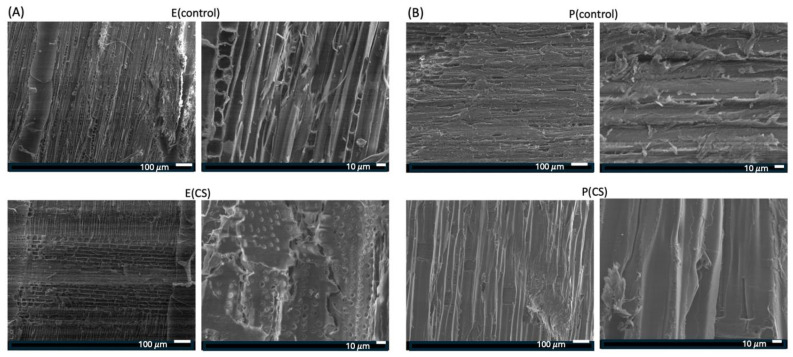
SEM images of *Eucalyptus* sp. (**A**) and *Pinus* sp. (scale bars of 10 and 100 μm) (**B**) samples are shown. E(control) uncoated *Eucalyptus* sp. and P(control) uncoated *Pinus* sp. correspond to uncoated wood samples, while E(CS) and P(CS) are chitosan-coated samples at 70 bar. CS: chitosan of ultra-high molar mass.

Figure 4 shows SEM images of *Eucalyptus* sp (Figure 4A) and *Pinus* sp. (Figure 4B) samples before and after CS coating. The surfaces of the control samples (E(control) and P(control)), which were not coated, appear rougher due to the preparation method of the test specimens (2 cm^3^), which were cut with a circular saw. Notably, there are visible pores in the uncoated E(control) sample. The CS-coated samples appear to have a smoother surface than the uncoated ones. The SEM image of the E(CS) sample shows partially filled pores, suggesting that the CS coating was successful, impregnating CS in the pore surfaces. The high pressure of 70 bar seems to have contributed to the deposition of CS on the samples and the filling of the pores, which is visible in the E(CS) sample. These results are consistent with the findings from EDS and XPS, confirming the deposition of CS on the wood surface.

The surface coatings based on CS and TN induce color parameter changes in the wood samples. Table 2 presents the measured color parameters, including clarity/brightness (L*), redness/greenness (a*), yellowness/bluishness (b*), total color difference (ΔE), and whiteness index (WI). All samples exhibit intermediate L* values, ranging from 44 ± 3 to 74 ± 3 (*p* ≤ 0.05). Elevated L* values indicate a colorless, luminous, and homogeneous material [31]. Changes in L* values occur after polymer deposition and surface coating formation with uncoated *Eucalyptus* sp. (L* = 60 ± 4, *p* ≤ 0.05) and *Pinus* sp. (L* = 74 ± 3, *p* ≤ 0.05) presenting the highest L* values.

The samples display positive a* parameters, with values lower for *Pinus* sp. samples than *Eucalyptus* sp., suggesting a slight greenish color in *Pinus* sp. samples. Additionally, the reddish color of *Eucalyptus* sp. occurs after surface modification. The b* parameter undergoes slight alterations for both wood samples after coating.

**Figure 5:**
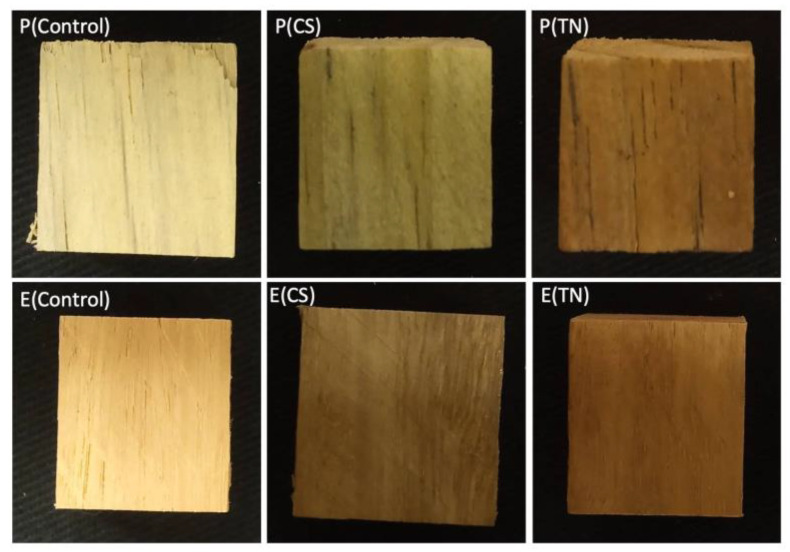
Digital images of the wood samples before and after surface modification with CS and TN at 70 bar. Sample labels include E(control): uncoated *Eucalyptus* sp.; P(control): uncoated *Pinus* sp.; E(CS): *Eucalyptus* sp. coated with CS; E(TN): *Eucalyptus* sp. coated with TN; P(CS): *Pinus* sp. coated with CS; P(TN): *Pinus* sp. coated with TN.

Digital images in Figure 5 show the color changes in the samples following surface modification with the polymers. The color slightly shifts towards greenish and brown hues due to the CS and TN surface coatings. The ΔE value for the coated wood ranges from 30 ± 2 to 53 ± 2 (*p* ≤ 0.05). Of note is that the human eye cannot discern color changes if the ΔE is less than 3.0 [32]. The whiteness index (WI) also exhibits statistically significant differences for the *Eucalyptus* sp. and *Pinus* sp. samples after surface coating deposition (*p* < 0.05).

The *Pinus* sp. and *Eucalyptus* sp. samples were also characterized by FTIR-ATR, with the spectra presented in the Supplementary Materials (Figures S4 and S5). The spectra of the control samples, i.e., those not coated with CS and TN, are practically identical to those of the coated samples. This result suggests that the CS predominantly deposits on the surface of the test specimens, as its high molecular weight and the viscosity of its solution hinder its penetration into the bulk of the wood. The presence of CS in internal regions likely occurs on the surface of the pores, as suggested by the SEM images (Figure 4).

Other factors that may influence these results include the small surface area that FTIR-ATR can analyze and the structural similarity between the main wood components (cellulose and hemicellulose) with the chemical structure of CS. All these materials exhibit similar connectivity, composed primarily of carbon and oxygen, making bulk material characterization challenging. However, CS, known for its film-forming properties, combined with the high pressure used in the coating process, supports the deposition of CS films impregnated into the pore surfaces of the wood samples. On the other hand, characterization through EDS, XPS, SEM, WCA, and FTIR-ATR suggests that the impregnation of the samples is superficial, thus justifying the term “coated wood samples”.

### 2.2. Fungicidal Activity of the Polymers against Neolentinus lepideus and Trametes versicolor

**Figure 6:**
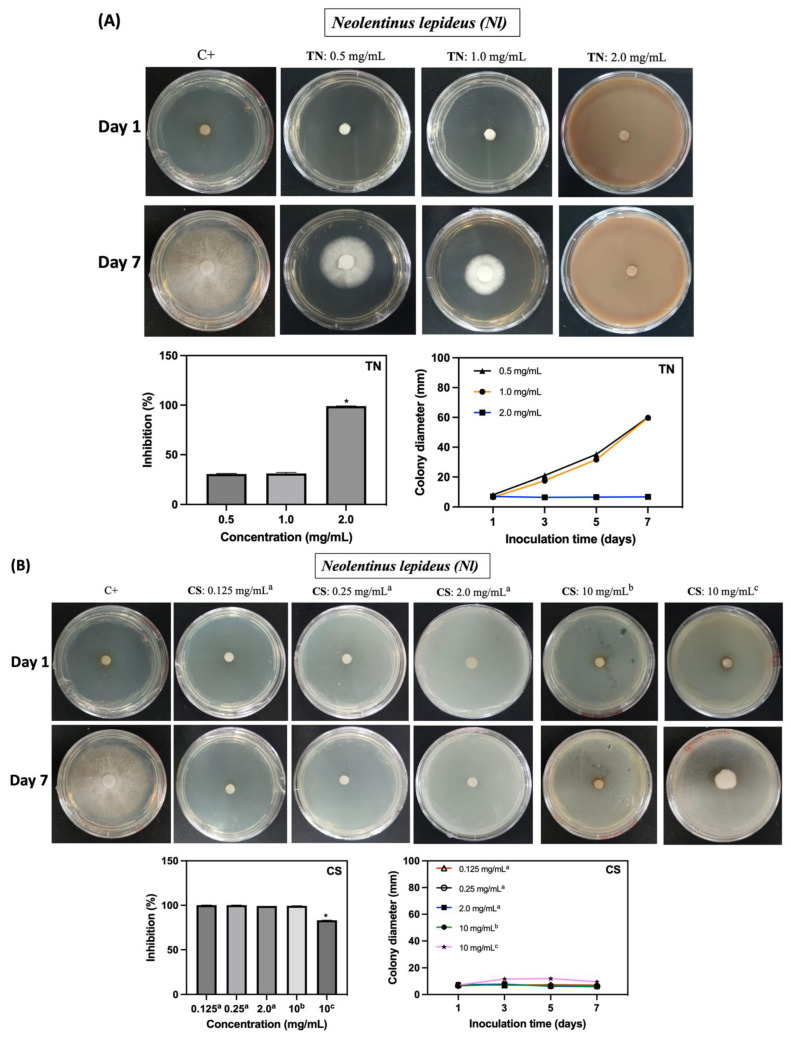

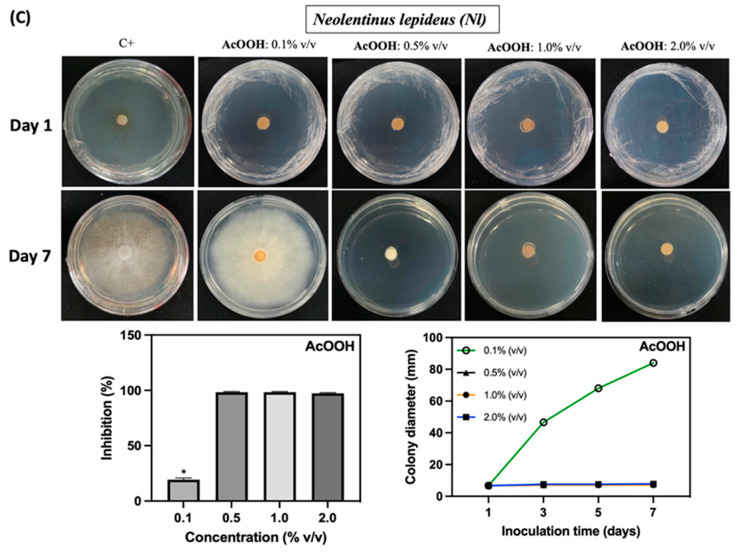
Inhibitory effect of the TN (**A**), CS (**B**), and AcOOH solutions (**C**) against *Neolentinus lepideus* (*Nl*) within 7 days of incubation. In the control assay (C+), the fungus is seeded in the Petri dish without CS, TN, and AcOOH. In (**B**), overwritten letters, including a, b, and c, indicate the concentration of AcOOH used to solubilize the CS (a = 1.0% *v*/*v*, b = 0.5% *v*/*v*, and c = 0.1% *v*/*v*, respectively. The term * indicates results with significant differences (*p* ≤ 0.05).

**Figure 7:**
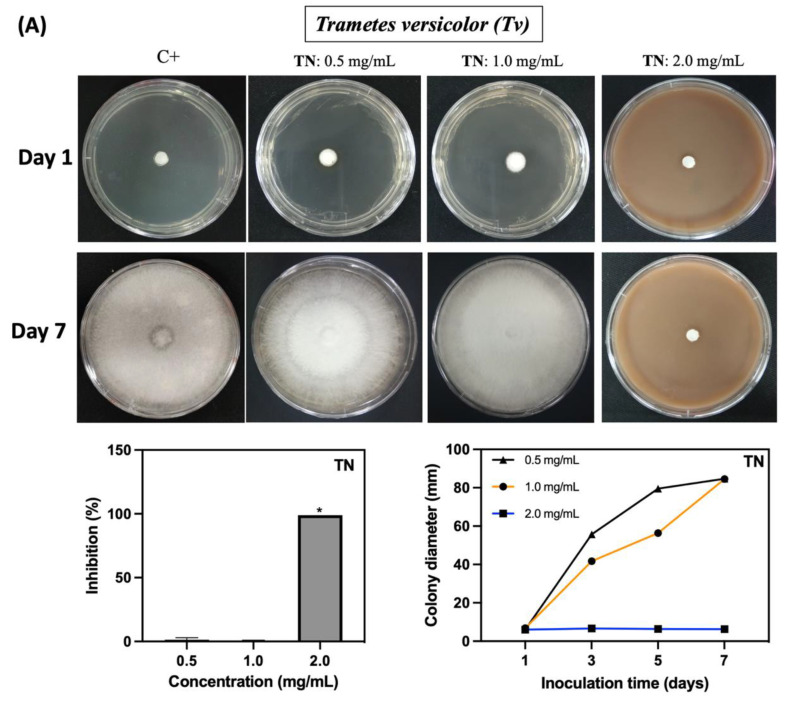

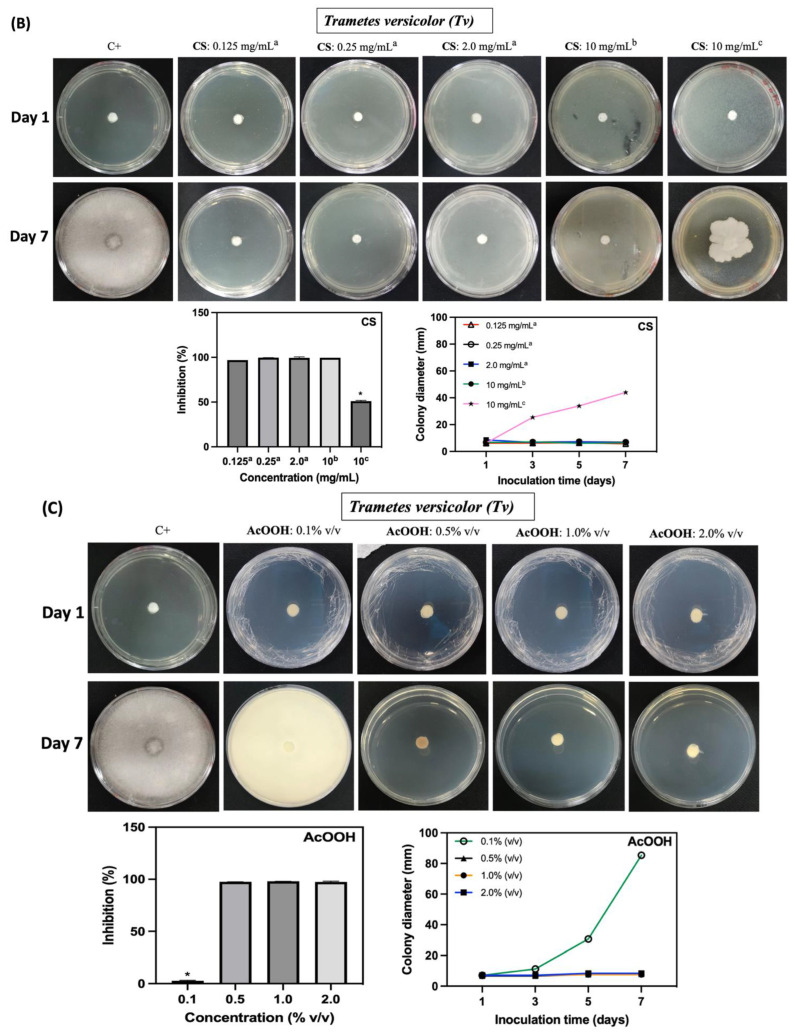
Inhibitory effect of the TN (**A**), CS (**B**), and AcOOH solutions (**C**) against *Trametes versicolor* (*Tv*) within 7 days of incubation. In the control assay (C+), the fungus is seeded in the Petri dish without CS, TN, and AcOOH. In (**B**), overwritten letters, including a, b, and c, indicate the concentration of AcOOH used to solubilize the CS. The letters a, b, and c in (**B**) represent AcOOH at 1.0, 0.5, and 0.1% *v*/*v*, respectively. The term * indicates results with significant differences (*p ≤* 0.05).

The fungicidal activity of CS, TN, and AcOOH solutions against *Nl* and *Tv* was assessed in vitro. Solution concentrations were adjusted to achieve fungicidal activity and inhibit the mycelial growth of both fungal species (Figure 6 and Figure 7). TN demonstrates significant inhibition of fungal growth at 2.0 mg/mL (*p* ≤ 0.05), resulting in an approximate 99% inhibition for both fungi (Figure 6A and Figure 7A). CS solutions, prepared in diluted AcOOH solutions, exhibited potent fungicidal activity. In a 1.0% *v*/*v* AcOOH solution, CS completely inhibits the growth of *Nl* and *Tv* from 0.0125 mg/mL and 0.125 mg/mL, respectively (Figure 6B and Figure 7B). The fungicidal concentrations for CS differ from TN, supporting the evaluation of the fungicidal activity of AcOOH aqueous solutions without CS. AcOOH inhibits both fungi growth at 0.5% *v*/*v* (Figure 6 and Figure 7). The difference in fungicidal concentrations explains the superior fungicidal activity of CS compared to TN. The presence of AcOOH in CS solution is a crucial factor influencing CS fungicidal activity.

The concentration of AcOOH significantly influences its efficacy against the *Nl* and *Tv* microorganisms. A concentration of 0.1% *v*/*v* (62.8 mg/mL) of AcOOH is ineffective in inhibiting fungal growth (Figure 6C and Figure 7C). A low concentration of CS (0.00125 mg/mL) completely suppresses fungal growth completely (Figure 6B and Figure 7B). In the control assay (C+), *Nl* and *Tv* hyphae display growth, while CS, TN, and AcOOH effectively prevent the mycelial growth of the fungi. A synergistic effect is noted between CS and AcOOH, inhibiting the fungi.

**Figure 8:**
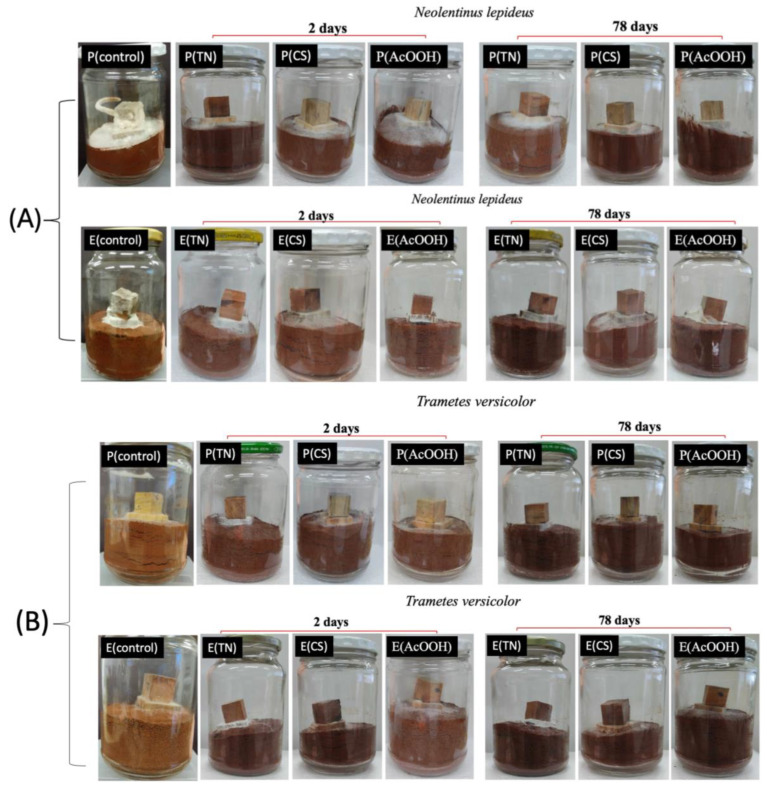
Digital images associated with the samples of *Pinus* sp. And *Eucalyptus* sp., which were incubated at 26 ± 2 °C and 70 ± 4% relative humidity. (**A**) Decay test with *Pinus* sp. and *Eucalyptus* sp. in the presence of isolates of *Neolentinus lepideus*, conducted over 78 days. The samples are identified as follows: P(control)—uncoated *Pinus* sp.; P(TN)—*Pinus* sp. coated with tannins; P(CS)—*Pinus* sp. coated with chitosan; P(AcOOH)—*Pinus* sp. treated with acetic acid; E(control)—uncoated *Eucalyptus* sp.; E(TN)—*Eucalyptus* sp. coated with tannins; E(CS)—*Eucalyptus* sp. coated with chitosan; E(AcOOH)—*Eucalyptus* sp. treated with acetic acid. (**B**) A decay test with *Pinus* sp. and *Eucalyptus* sp. in the presence of isolates of *Trametes versicolor* was also conducted over 78 days, with the same nomenclature for the samples.

In contrast, TN exhibits no inhibitory effect at concentrations below 2.0 mg/mL. Fungal growth is evident in the Petri dish containing 10 mg/mL of CS due to the low concentration of AcOOH used to solubilize CS (0.1% *v*/*v*). The digital images in Figure 6 and Figure 8 illustrate the growth of fungi under these conditions.

The crucial role of AcOOH in solubilizing CS is evident, as CS neutralizes AcOOH in solution. The amino groups of CS are partially in an AcOOH aqueous solution. Investigating the fungicidal activity of AcOOH confirms the fungicidal potential of CS, given that CS has solubility in organic acid aqueous solutions. These findings align with previous studies. Kothari and Lee [33] reported a 10% reduction in the cellular activity of *Escherichia coli* when in contact with an AcOOH aqueous solution at 1.0 g/L. Furthermore, an AcOOH solution at pH 3.4 effectively killed *Aspergillus flavus* [34]. AcOOH exhibits cytotoxicity against various yeasts, fungi, and bacteria [35,36,37,38].

CS has outstanding antimicrobial activity against bacteria and fungi [39,40,41,42] and effectively inhibits the growth of *Nl* and *Tv*. Research indicates that the fungicidal activity of CS depends on factors such as its deacetylation degree, molar mass, and pH [43]. The antimicrobial activity of CS is attributed to coulombic interactions established with cellular membranes rich in negatively charged phospholipids. This interaction can result in the permeabilization of the fungal plasma membrane, releasing intracellular constituents and ultimately causing the death of the pathogen [44]. Consequently, ionized CS containing -NH_3_^+^ groups has been shown to inhibit the growth of fungi and bacteria [18,45], including *Candida albicans* and *S. aureus*, respectively [46,47]. Furthermore, CS presents antimicrobial activity against microorganisms resistant to antibiotics (vancomycin, methicillin, fluconazole, amphotericin B, and caspofungin) [46,48]. However, CS supports cytocompatibility for human cells [40].

CS exhibits fungicidal effects, particularly against various phytopathogenic fungi, with notable activity against soil-dwelling fungi such as the pathogen *Fusarium* [49]. Its ability to permeate the fungal cell membrane allows it to act on intracellular components, disrupting the physiological processes of the fungus. Furthermore, CS can directly bind to fungal genetic materials, suppressing DNA replication and inducing the death of the pathogen [44,50]. Ren and collaborators [51] showed that 0.215 g/L of CS (with a deacetylation degree higher than 90%) in 1.0% *v*/*v* AcOOH inhibited the growth of spores of the phytopathogen *Fusarium oxysporum*, which causes dry rot in potatoes.

Various mechanisms have been proposed to explain the antifungal action of CS. Among them, notable mechanisms include localized rupture of fungal cell membranes, cytoplasmic leakage, chelation of essential nutrients, and direct interaction with nucleic acids, leading to alterations in genetic information flow and interrupting the replication of fungal genetic material [52]. Several factors, such as molecular weight, degree of deacetylation, the type of microorganism, and environmental conditions, including ionic strength and pH, influence the antimicrobial efficacy of CS [53]. The molecular weight of CS directly affects its mode of action on microbial cells. High molecular weight CS tends to adhere to the cell surface, forming a layer that weakens and ruptures cell walls, leading to the loss of intracellular content. In contrast, low molecular weight CS can penetrate cells, where they bind to DNA, inhibit enzyme activity, and interrupt protein synthesis, resulting in cell death [54,55].

TN comprises hydrolyzable and primarily condensed tannins [56]. Gallic acid (phenolic acid) and other phenolic compounds have been shown to inhibit mycelial fungi by accumulating within the microorganism [57]. Additionally, tannins can inhibit fungal enzymes by forming complexes, disrupting metabolic processes, and preventing the development of microorganisms [58]. The antimicrobial properties of tannins are attributed to their complex and heterogeneous chemical compositions, which include flavonoids, alkaloids, and gallic acid-based compounds [59].

### 2.3. Antifungal Activity In Vivo

Table 3 presents the mass gain in wood samples following the deposition of TN and CS coatings and treatment with AcOOH. The control tests confirmed a high water absorption and retention in the samples, with a 73% increase in mass after pressurization in distilled water for *Pinus* sp. and 42% for *Eucalyptus* sp. (Table 3). However, the mass gain after the pressurization of the wood samples in the solutions was slightly lower for the samples incubated with CS and TN compared to the control samples and those impregnated with AcOOH. This result may be associated with the properties of the polymeric solutions, which, due to their higher viscosity, have more difficulty penetrating the internal structure of the wood. These results suggest that the CS coating should comprise a thin film layer on the wood samples.

Statistical analysis indicates that the mass gain is independent of the type of polymer (CS and TN) used in the coating (*p* > 0.05). A more substantial mass gain is evident in *Pinus* sp. compared to *Eucalyptus* sp. This finding is attributed to the density of the wood. Less dense wood possesses more empty spaces in its bulk, allowing higher amounts of water, CS, TN, and AcOOH deposition. Consequently, there is a more significant mass gain after coating with CS and TN in *Pinus* sp. than in *Eucalyptus* sp.

Figure 8 shows digital images of the uncoated (control), TN- or CS-coated, and AcOOH-treated wood samples seeded on feeding substrates contaminated with *Nl* or *Tv*. The digital images of the uncoated samples (P(control) and E(control)) were captured over 78 days of incubation (Figure 8). Significantly, fungi growth is observed on the control samples without polymer coatings. Furthermore, samples treated with AcOOH do not prevent fungi deposition and growth (Figure 8). However, samples coated with TN and CS effectively inhibit the growth of fungi responsible for biodeterioration over the 78 days of incubation (Figure 8).

The surface coatings exhibit a substantial preventive effect against fungal contamination, primarily attributed to the formation of polymeric films on the wood samples. These films act as effective barriers, preventing the deposition and contamination of fungi. The antimicrobial activity inherent in CS and TN further supports the protective capacity. However, no significant mass change was observed in uncoated and coated samples after 78 days of incubation with the fungi. The exposure time to fungi is relatively short. Therefore, extended exposure times could indicate prominent changes in the mass of control samples contaminated with fungi compared to those treated with AcOOH and coated with polymers.

### 2.4. Leaching

Table 4 presents the percentage of leached mass from the control samples (E(control) and P(control)) and the samples coated with CS and TN. The coated materials exhibit a significantly lower leached mass than the mass leached from the controls, indicating that the coatings stabilize the wood by reducing the leaching of potential additives. A higher concentration of additives seems to be released from the control wood samples (Table 4). CS and TN thin films deposited on the wood samples should reduce the diffusion rate of water molecules into the wood bulk, reducing the leaching of low-molecular-weight additives.

**Figure 9:**
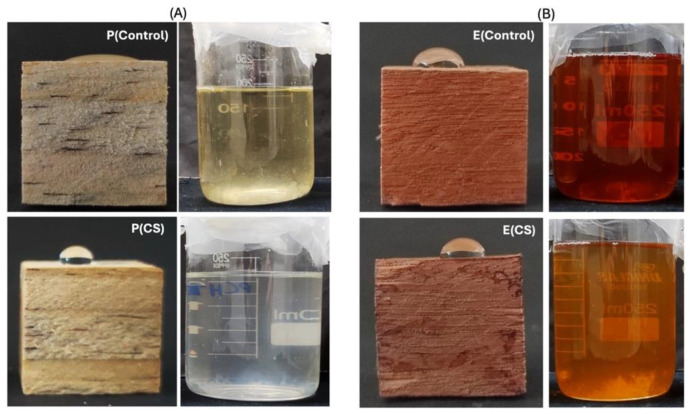
Digital images of the wood samples with a drop of water on the surface, taken after the leaching test (**A**). Digital images of the supernatant water that remained in contact with the wood samples after the leaching test (**B**). E(control) uncoated *Eucalyptus* sp.; P(control) uncoated *Pinus* sp.; P(CS): *Pinus* sp. coated with CS; E(CS): *Eucalyptus* sp. coated with CS.

Figure 9 presents digital images of the wood samples (controls and CS-coated ones) after the leaching test. The *Pinus* sp. coated with CS still exhibits hydrophobicity. This result is confirmed by the digital image in Figure 9A (left panel), where a drop of water only spreads over the control sample surface. This finding indicates that the coating remained on the surface of *Pinus* sp. after the leaching test. In contrast, the result appears to be different for the *Eucalyptus* sp., as no visible difference in wettability is observed on the surface of the *Eucalyptus* sp. samples (uncoated control and E(CS)) after the leaching test (Figure 9B, right panel).

The brown coloration of the supernatant water indicates the leaching of additives. This color is more intense in the supernatant from the control samples, suggesting the leaching of phenolic compounds, such as lignocellulosic components and tannins, particularly from *Eucalyptus* sp. In contrast, the *Pinus* sp. coated with CS displays clear and colorless supernatant water, indicating that no significant leaching of additives or other materials occurred from the coated *Pinus* sp. (Figure 9A).

The hydrophobic CS coatings proposed in this study may have limitations. Previously published results showed that CS does not exhibit stability and durability when in contact with soil. Wrońska et al. [60] observed that CS biodegraded after two weeks of direct contact with soil. This study used a feeding substrate to separate the coated wood samples from the soil, preventing direct contact. On the other hand, there are outcomes indicating that the degradation rate of CS in the soil varies from a few weeks to several months depending on soil type, environmental conditions, and CS properties, including molecular weight, degree of acetylation, source, etc. [61].

Munstermann et al. [62] showed the effectiveness of CS coatings associated with itaconate in wood protection. After four weeks of exposure to irradiation, the coating protected the wood against UV light degradation and effectively prevented fungal growth.

## 3. Materials and Methods

### 3.1. Materials

Chitosan (CS), with an ultra-high molar mass of 145 × 10^5^ g/mol, was purchased from Golden-Shell Biochemical (Shanghai, China). Tannin (TN) extracts from Acacia decurrens De Wild, commonly known as Acácia Negra, were provided by Tanac SA (Montenegro-RS, Brazil) and used as a control sample due to their antifungal activity and numerous reports highlighting their effectiveness as wood preserving agents [4,13,14,63]. The glacial acetic acid (AcOOH) was obtained from Synth (São Paulo, Brazil), and its diluted aqueous solutions were also used as control samples for wood preservation, as these solutions are commonly used to dissolve CS [64].

### 3.2. Surface Coatings Deposited on Wood through Ultra-Pressurization

The coating deposition involved ultra-pressurizing wood samples in contact with CS, TN, or diluted acetic acid (AcOOH) aqueous solutions using the equipment outlined in the patent by Filho and coworkers [65]. The apparatus provides mechanical pressurization and consists of a rectangular stainless-steel compartment. *Eucalyptus* sp. and *Pinus* sp. samples (2 cm^3^) of known mass were placed into this compartment with CS or TN aqueous solutions at 1.0% *w*/*v* or diluted AcOOH aqueous solutions without polymers (Table 5). CS aqueous solutions were prepared in diluted AcOOH aqueous solutions, while the TN aqueous solutions were yielded in distilled water (Table 5). Subsequently, five wood samples were soaked in the solutions (95 mL), and the compartment containing the solutions and wood samples was sealed using a metal base secured with 8 screws. The system was subjected to a pressure of 70 bar for 1 h at room temperature. Following the ultra-pressurization process, the samples were removed from the metallic compartment and rinsed once with distilled water to eliminate any excess material from the surfaces. They were then oven-dried at 50 °C for 72 h and weighed to determine the mass gained after coating using Equation (1):

(1) $$Mass\;gain\left(\%\right)=\left(\frac{M_{2}-M_{1}}{M_{1}}\right)\times 100$$

where the mass gain (%) is calculated based on the mass alteration of the samples following the wood treatment. Here, M_1_ represents the samples’ initial mass (g) before exposure to ultra-pressurization, and M_2_ represents the mass (g) of the samples after ultra-pressurization with the CS, TN, or AcOOH solution. After quantifying the mass gain, the samples were stored in a desiccator for further analysis.

### 3.3. Characterization

CS and TN (precursors) were characterized through Fourier transform infrared spectroscopy using a Shimadzu Scientific Instrument (Cary 630, Columbia, MD, USA) (Supplementary Materials, Figure S6). The spectra were obtained in attenuated total reflectance mode (FTIR-ATR) with a resolution of 4 cm^−1^ and 64 scans. Dynamic light scattering (DLS) measurements were conducted with the CS and TN aqueous solutions using a LiteSizer 500 instrument (Anton Paar, Luton, Bedfordshire, UK) [66]. The solutions’ Zeta potential and hydrodynamic radius were measured at room temperature. These results are presented in the Supplementary Materials, while the chemical structures of CS and TN are well-established and documented in the literature [64,67].

FTIR-ATR spectra were also obtained from the surfaces of the wood samples before and after treatment by ultra-pressurization with CS and TN solutions, using the methodology and equipment described previously. Water contact angle (WCA) measurements were evaluated on the surfaces of dried samples at 25 °C. The assessments were conducted with water on coated and uncoated wood samples, using the sessile drop method and a contact angle goniometer Krüss DAS 10 (Hamburg, Germany), equipped with video capture. A water droplet (3.0 µL) was deposited on the sample surfaces, and three measurements were taken on each sample in various areas [68]. Digital images of the surfaces were captured and correlated with the WCA values.

The surface chemical composition of both coated and uncoated samples was characterized through X-ray excited photoelectron spectroscopy (XPS). XPS survey spectra were captured using Phi Electronics 5800 Spectrometer equipment (Chanhassen, MN, USA) coupled with a monochromatic Al Kα X-ray source (hν = 1486.6 eV), employing a hemispherical analyzer and a multichannel detector. High-resolution XPS spectra were obtained using a 23.5 eV pass energy analyzer with 0.10 eV steps and an 800 μm X-ray spot. The spectra were fitted using Origin version 8.5, employing a Shirley background. The spectra were acquired with a photoelectron takeoff angle of 45° [69].

Samples of *Eucalyptus* sp. and *Pinus* sp., both coated and uncoated, were characterized through scanning electron microscopy (SEM) and elemental mapping using energy-dispersive X-rays using a JSM-6500F electron microscope (JEOL, Tokyo, Japan) operating at an accelerating voltage of 5 kV. Before obtaining SEM images, a thin layer of palladium–gold alloy (10 nm) was deposited on the samples using a Polaron SC 7620 Sputter Coater (Quorum Technologies, Newhaven, UK) [17]. Elemental mapping for carbon, oxygen, and nitrogen was carried out on the surfaces coated with CS using the JSM-6500F instrument [69].

A Deltra Vista spectrophotometer (Brazil) was used to analyze the color of the samples before and after coating. The parameters of lightness/brightness (L*), redness/greenness (a*), and yellowness/blueness (b*) were evaluated and used to obtain the parameters color difference (ΔE) and the whiteness index (WI) through Equations (2) and (3), respectively [70]:

(2) $$∆E=\sqrt{(L*-{L)}^{2}+(a*-{a)}^{2}+(b*-{b)}^{2}}$$

(3) $$WI=100-\sqrt{(100-{L)}^{2}+a^{2}+b^{2}}$$

### 3.4. Antimicrobial Tests with Wood Rot-Causing Fungi

#### 3.4.1. Fungal Material

The fungal strains used in this study, *Neolentinus lepideus* (*Nl*, Mad-534) and *Trametes versicolor* (*Tv*, CCIBT 2539), were obtained from the United States Department of Agriculture (USDA)/Forest Service (FS)/Forest Products Laboratory (FPL), Madison, WI, USA, and Botany Institute, São Paulo, Brazil, respectively. The isolates were selected and cultured in Petri dishes on potato, dextrose, and agar medium (PDA) with a pH of 5.6. The cultures were incubated at 25 ± 2 °C in a Biochemical Oxygen Demand incubator and stored for 7 days for subsequent use.

#### 3.4.2. In Vitro Antifungal Activity of Biopolymers and Acetic Acid Solutions

CS, TN, and AcOOH aqueous solutions were prepared and autoclaved at 55 °C. CS solutions were obtained in diluted AcOOH aqueous solutions (Table 5). Subsequently, aliquots of CS, TN, and AcOOH solutions were added to the PDA medium and autoclaved. *Tv* and *Nl* isolates with a mycelium diameter of 8.0 mm were inoculated in Petri dishes containing 20 mL of BDA with antifungal agents at different concentrations (Table 5).

The Petri dishes were placed in a growth chamber at 25 ± 2 °C for 7 days. The experiment was evaluated in triplicate for each isolate. The diameter of mycelial growth was measured with a digital caliper. The growth inhibition rate of *Tv* and *Nl* fungi was determined using Equation (4) [71].

(4) $$Inihibitory\;rate\left(\%\right)=\left[\left(C-T\right)\right]/\left[\left(C-0.6\right)\right]\times 100$$

where C is the diameter of the control halo (0.6 cm), and T is the halo of the hyphae (cm) of the fungi after contact with the PDA medium containing the antifungal agents (CS, TN, or AcOOH solutions) determined over 7 days of analysis with the caliper.

#### 3.4.3. In Vivo Preservative Treatment of Wood

*Pinus* sp. and *Eucalyptus* sp. samples (2 cm^3^) were obtained from Empório da Madeira (Maringá, Brazil). The test samples were sanded, and those without knots and cracks were selected for testing. Subsequently, they were placed in an oven at 50 °C for 72 h until reaching a constant mass.

Rot tests with adaptations were evaluated following the methodology outlined in the American Wood Protection Association standard (AWPA E10-22) [71]. Samples of purple-dystrophic oxisol (200 g, pH of 6.0, and relative humidity of 130%), without organic matter and with a particle size of 600 µm, were added to glass bottles (500 mL) with screw caps. The soil moisture (130%) was adjusted using Equation (5) by calculating the amount of distilled water necessary to achieve the desired relative humidity.

(5) $$A_{H_{2}o}=\left[1.30\times \left(A-B\right)\right]\times \left[M_{soil}∕\left(100+B\right)\right]$$

where $A_{H_{2}o}$ is the determined mass (g) of distilled water added to each flask (80 g), A is the water retention capacity of the soil (%), B is the moisture content of the dry soil (%), and $M_{soil}$ is the mass of dry soil (200 g) added to each flask.

*Pinus* sp. or *Eucalyptus* sp. sample (0.5 × 3.0 × 3.0 cm^3^) was positioned on the soil previously added to each flask. These samples served as a substrate for the growth of Nl and Tv fungi. Subsequently, the flasks were autoclaved at 121 ± 1 °C for 45 min. Each flask received a 6 mm disk of mycelium from Nl or Tv fungal isolates deposited on the wood substrates. The flasks were then incubated at 26 ± 2 °C and 70 ± 4% relative humidity for 45 days to allow the mycelia to cover the substrates entirely.

After the feeder substrates were covered with fungi, the uncoated and coated wood samples (2 cm^3^) were placed on the feeder substrates containing Nl or Tv. The system was incubated for 12 weeks at 26 ± 2 °C and 70 ± 4% relative humidity. Afterward, the samples were removed from the glass bottles and stored in a ventilated oven at 50 °C for 72 h until they reached constant masses. The mass reduction (%) was determined using Equation (6).

(6) $$Mass\;reduction\left(\%\right)=\left(\frac{M_{A}-M_{B}}{M_{B}}\right)\times 100$$

where $M_{A}$ is the initial mass (g) of the samples before exposure to fungi (after pressurization with the CS, TN, and AcOOH solutions), and $M_{B}$ is the mass (g) of the samples after the rotting test.

### 3.5. Leaching Assay

The leaching assay followed a previously reported experimental procedure [16] with modifications. Ten coated and uncoated samples (2 cm^3^) of *Eucalyptus* sp. and *Pinus* sp. were placed in 250 mL flasks containing 150 mL of distilled water and incubated on a shaker (Solab) without orbital agitation at room temperature for 15 days at room temperature. After 15 days, the samples were removed from the water and oven-dried at 50 °C for 72 h, following the guidelines outlined in the American Wood Protection Association standard (AWPA E10-22) [71]. The leaching (%) of CS and TN was determined using Equation (7). The leached mass (%) results were compared with the results for the control sample (uncoated), which was previously soaked in distilled (15 days) and then dried.

(7) $$Leachead\;mass\left(\%\right)=\left(\frac{M_{A}-M_{C}}{M_{C}}\right)\times 100$$

The $M_{A}$ term is the initial mass (g) of the samples before exposure to fungi (after ultra-pressurization with the CS and TN solutions), and $M_{C}$ is the mass (g) of the samples after the leaching assay. The wettability of the samples obtained after the leaching assay was investigated by depositing a water droplet (10 μL) on the surface of the dried samples. Digital images of the water in contact with the samples were taken after 15 days, and the supernatant water in contact with the samples (uncoated and coated) was compared.

### 3.6. Statistical Analysis

The results were statically analyzed through ANOVA analysis and the Tukey test, with a significance level of 5% using GraphPad Prism 8.0.

## 4. Conclusions

This study introduces a novel method to develop surface coatings based on chitosan (CS) on wood samples using ultra-pressurization, a strategy involving mechanical pressure applied between polymer aqueous solutions and wood substrates. In vitro antimicrobial tests confirm the outstanding antimicrobial activity of both TN and CS against *Trametes versicolor* (*Tv*) and *Neolentinus lepideus* (*Nl*). In vivo antimicrobial assays further ratify their efficacy, underscoring the substantial protective nature of CS and TN coatings against *Eucalyptus* sp. and *Pinus* sp. This pioneering study presents a promising wood preservation strategy, highlighting the potential of CS as a natural bio-fungicide. This approach provides an environmentally friendly alternative to traditional chemical fungicides. The comprehensive inhibitory effect of CS on various phytopathogenic fungi can hold significant implications for sustainable practices, reducing reliance on synthetic chemicals.

## Figures and Tables

**Table 1:** Water contact angles (WCAs) measured on the wood samples.

Samples	WCAs (°)		
	0 s	3 s	5 s
E(Control)	79 ± 1 ^a^	79 ± 1 ^a^	79 ± 1 ^a^
E(CS)	118 ± 1 ^b^	118 ± 1 ^b^	118 ± 1 ^b^
E(TN)	80 ± 1 ^c^	89 ± 1 ^a^	86 ± 1 ^a^
E(AcOOH)	54 ± 1 ^d^	45 ± 1 ^c^	41 ± 1 ^c^
P(Control)	17 ± 10 ^a′^	17 ± 10 ^a′^	17 ± 10 ^a′^
P(CS)	124 ± 1 ^b′^	124 ± 1 ^b′^	123 ± 1 ^b′^
P(TN)	80 ± 1 ^c′^	68 ± 1 ^c′^	61 ± 1 ^c′^
P(AcOOH)	0 ^d′^	0 ^d′^	0 ^d′^

P(Control): uncoated *Pinus* sp.; P(AcOOH): *Pinus* sp. coated with the AcOOH; P(TN): *Pinus* sp. coated with TN; P(CS): *Pinus* sp. coated with CS; E(Control): uncoated *Eucalyptus* sp.; E(AcOOH): *Eucalyptus* sp. coated with AcOOH; E(TN): *Eucalyptus* sp. coated with TN; E(CS): *Eucalyptus* sp. coated with CS. Results are presented as mean ± standard deviation; *n* = 3. Different letters in the same column indicate results with significant differences (*p* ≤ 0.05). The notation is used to statistically compare the results between different species. The letters without the prime are used to represent results for *Eucalyptus* sp. samples, while those with the prime indicate the corresponding results for *Pinus* sp.

**Table 2:** Color parameters of the uncoated and coated wood samples.

Samples	L*	a*	b*	ΔE	WI
E(Control)	60 ± 4 ^a^	13 ± 4 ^a^	22 ± 3 ^a^	43 ± 4 ^a^	52 ± 4 ^a^
E(AcOOH)	54 ± 6 ^b^	13 ± 1 ^a^	19 ± 7 ^b^	46 ± 6 ^a,b^	50 ± 7 ^a^
E(CS)	44 ± 3 ^c^	12 ± 2 ^a^	13 ± 1 ^c^	53 ± 2 ^c^	41 ± 3 ^b^
E(TN)	50 ± 4 ^d^	13 ± 2 ^a^	15 ± 0 ^b,c^	49 ± 3 ^b^	45 ± 3 ^c^
P(Control)	74 ± 3 ^a′^	5 ± 2 ^a′^	21 ± 2 ^a′^	30 ± 2 ^a′^	66 ± 2 ^a′^
P(AcOOH)	66 ± 4 ^b′^	4 ± 0 ^a′^	17 ± 2 ^b′^	33 ± 2 ^a′^	62 ± 3 ^b′^
P(CS)	59 ± 6 ^c′^	5 ± 1 ^a′^	18 ± 2 ^c′^	40 ± 5 ^b′^	54 ± 5 ^c′^
P(TN)	53 ± 5 ^d′^	11 ± 2 ^b′^	16 ± 1 ^b′^	45 ± 4 ^c′^	49 ± 4 ^d′^

E(control): uncoated *Eucalyptus* sp.; P(control): uncoated *Pinus* sp.; E(CS): *Eucalyptus* sp. coated with CS; E(TN): *Eucalyptus* sp. coated with TN; E(AcOOH): *Eucalyptus* sp. treated with AcOOH; P(CS): *Pinus* sp. coated with CS; P(TN): *Pinus* sp. coated with TN; P(AcOOH): *Pinus* sp. treated with AcOOH. Results are presented as mean ± standard deviation; *n* = 3. Different letters in the same column indicate results with significant differences (*p* ≤ 0.05). The notation is used to statistically compare the results between different species. The letters without the prime are used to represent results for *Eucalyptus* sp. samples, while those with the prime indicate the corresponding results for *Pinus* sp.

**Table 3:** Mass gain after coating deposition (TN or CS) and treatment with AcOOH.

Mass Gain (%)							
P(control)	P(CS)	P(TN)	P(AcOOH)	E(control)	E(CS)	E(TN)	E(AcOOH)
73 ± 5	63 ± 2	63 ± 5	67 ± 1	42 + 4	38 ± 5	34 ± 6	41 ± 16

E(control): uncoated *Eucalyptus* sp.; P(control): uncoated *Pinus* sp.; E(CS): *Eucalyptus* sp. coated with CS; E(TN): *Eucalyptus* sp. coated with TN; E(AcOOH): *Eucalyptus* sp. treated with AcOOH; P(CS): *Pinus* sp. coated with CS; P(TN): *Pinus* sp. coated with TN; P(AcOOH): *Pinus* sp. treated with AcOOH. Results are presented as mean ± standard deviation; *n* = 3.

**Table 4:** Leached mass from wood samples after the stability test.

Samples	Leached Mass (%)
E(Control)	9.0 ± 0.2 ^a^
E(CS)	7 ± 1 ^b^
E(TN)	6.5 ± 0.9 ^b^
P(Control)	8.4 ± 0.2 ^a′^
P(CS)	7.2 ± 0.4 ^b′^
P(TN)	7.0 ± 0.5 ^b′^

E(control): uncoated *Eucalyptus* sp.; P(control): uncoated *Pinus* sp.; E(CS): *Eucalyptus* sp. coated with CS; E(TN): *Eucalyptus* sp. coated with TN; P(CS): *Pinus* sp. coated with CS; P(TN): *Pinus* sp. coated with TN. Results are presented as mean ± standard deviation; *n* = 3. Different letters in the same column indicate results with significant differences (*p* ≤ 0.05). The notation is used to statistically compare the results between different species. The letters without the prime are used to represent results for *Eucalyptus* sp. samples, while those with the prime indicate the corresponding results for *Pinus* sp.

**Table 5:** CS, TN, and AcOOH solutions used in antifungal assays against *Tv* and *Nl*.

Chemical Solutions	Concentration (mg/mL)	AcOOH Concentration (%*v*/*v*)
CS	5.0	1.0
	2.0	1.0
	0.25	1.0
	0.125	1.0
	10	0.1
	10	0.5
TN	2.0	*
	0.5	*
	0.1	*
AcOOH	2.1	2.0
	1.1	1.0
	0.52	0.5
	0.11	0.1
	0.011	0.01

CS: chitosan; TN: tannin; AcOOH: acetic acid. * TN aqueous solutions do not contain AcOOH (acetic acid) as TN is soluble in water.

## Data Availability

The data presented in this study are available upon request from the corresponding author due to privacy or ethical restrictions.

## Supplementary Materials

The following supporting information can be downloaded at: https://www.mdpi.com/article/10.3390/ijms252010899/s1.

## References

[1] Bossardi Dias K., Marques Barreiros R., Gong M. (2022). Preservative Treatments on Wood and Their Effects on Metal Fasteners. Engineered Wood Products for Construction.

[2] De Avila Delucis R., Gonzales De Cademartori P.H., Missio A.L., Alberto Gatto D. (2016). Decay Resistance of Four Fast-Growing Eucalypts Wood Exposed to Three Types of Fields. Maderas Cienc. Tecnol..

[3] Barnes H.M. (2001). Wood: Preservative Treated. Encyclopedia of Materials: Science and Technology.

[4] Barbero-López A., Akkanen J., Lappalainen R., Peräniemi S., Haapala A. (2021). Bio-Based Wood Preservatives: Their Efficiency, Leaching and Ecotoxicity Compared to a Commercial Wood Preservative. Sci. Total Environ..

[5] Lowden L., Hull T. (2013). Flammability Behaviour of Wood and a Review of the Methods for Its Reduction. Fire Sci. Rev..

[6] Jain S.H., Nagaveni H.C., Vijayalakshmi G. (2011). Effect of Leaf and Bark Extracts of *Cleistanthus collinus* (Benth. & Hook) and *Prosopis juliflora* (Sw.) DC in Combination with Inorganic Compounds against Wood Decay Fungi. J. Indian Acad. Wood Sci..

[7] Tascioglu C., Yalcin M., Sen S., Akcay C. (2013). Antifungal Properties of Some Plant Extracts Used as Wood Preservatives. Int. Biodeterior. Biodegrad..

[8] Yildiz Ü.C., Kilic C., Gürgen A., Yildiz S. (2020). Possibility of Using Lichen and Mistletoe Extracts as Potential Natural Wood Preservative. Maderas Cienc. Tecnol..

[9] Brocco V.F., Paes J.B., Costa L.G.D., Brazolin S., Arantes M.D.C. (2017). Potential of Teak Heartwood Extracts as a Natural Wood Preservative. J. Clean. Prod..

[10] Woźniak M., Kwaśniewska-Sip P., Waśkiewicz A., Cofta G., Ratajczak I. (2020). The Possibility of Propolis Extract Application in Wood Protection. Forests.

[11] Barbero-López A. (2021). Antifungal Activity of Several Vegetable Origin Household Waste Extracts Against Wood-Decaying Fungi In Vitro. Waste Biomass Valorization.

[12] Singh T., Singh A.P. (2012). A Review on Natural Products as Wood Protectant. Wood Sci. Technol..

[13] Sen S., Tascioglu C., Tırak K. (2009). Fixation, Leachability, and Decay Resistance of Wood Treated with Some Commercial Extracts and Wood Preservative Salts. Int. Biodeterior. Biodegrad..

[14] Tomak E.D., Gonultas O. (2018). The Wood Preservative Potentials of Valonia, Chestnut, Tara and Sulphited Oak Tannins. J. Wood Chem. Technol..

[15] Broda M. (2020). Natural Compounds for Wood Protection against Fungi—A Review. Molecules.

[16] Eikenes M., Alfredsen G., Christensen B.E., Militz H., Solheim H. (2005). Comparison of Chitosans with Different Molecular Weights as Possible Wood Preservatives. J. Wood Sci..

[17] Martins A.F., Vlcek J., Wigmosta T., Hedayati M., Reynolds M.M., Popat K.C., Kipper M.J. (2020). Chitosan/Iota-Carrageenan and Chitosan/Pectin Polyelectrolyte Multilayer Scaffolds with Antiadhesive and Bactericidal Properties. Appl. Surf. Sci..

[18] Kaya M., Baran T., Erdoğan S., Menteş A., Özüsağlam M.A., Çakmak Y.S. (2014). Physicochemical comparison of chitin and chitosan obtained from larvae and adult Colorado potato beetle (*Leptinotarsa decemlineata*). Mater. Sci. Eng. C.

[19] Goy R.C., Britto D.D., Assis O.B.G. (2009). A Review of the Antimicrobial Activity of Chitosan. Polímeros.

[20] El-Gamal R., Nikolaivits E., Zervakis G.I., Abdel-Maksoud G., Topakas E., Christakopoulos P. (2016). The Use of Chitosan in Protecting Wooden Artifacts from Damage by Mold Fungi. Electron. J. Biotechnol..

[21] Alorbu C., Cai L. (2022). Fungal Resistance and Leachability of Genipin-Crosslinked Chitosan Treated Wood. Int. Biodeterior. Biodegrad..

[22] Vilsinski B.H., De Oliveira A.C., Souza P.R., Martins A.F. (2024). Polysaccharide-Based Polyelectrolyte Multilayers Fabricated via Layer-by-Layer Approach: From Preparation to Applications. Prog. Org. Coat..

[23] Yordanov D.G., Angelova G.V. (2010). High Pressure Processing for Foods Preserving. Biotechnol. Biotechnol. Equip..

[24] Dumay E., Chevalier-Lucia D., Picart-Palmade L., Benzaria A., Gràcia-Julià A., Blayo C. (2013). Technological Aspects and Potential Applications of (Ultra) High-Pressure Homogenisation. Trends Food Sci. Technol..

[25] Li Y., Bao L., Song B., Han J., Li H., Zhao F., Liu H. (2013). A New Benzoquinone and a New Benzofuran from the Edible Mushroom Neolentinus Lepideus and Their Inhibitory Activity in NO Production Inhibition Assay. Food Chem..

[26] Schwarze F.W.M.R. (2007). Wood Decay under the Microscope. Fungal Biol. Rev..

[27] Zhang J., Meng Markillie L., Mitchell H.D., Gaffrey M.J., Orr G., Schilling J.S. (2022). Distinctive Carbon Repression Effects in the Carbohydrate-Selective Wood Decay Fungus Rhodonia Placenta. Fungal Genet. Biol..

[28] Sabino R.M., Mondini G., Kipper M.J., Martins A.F., Popat K.C. (2021). Tanfloc/Heparin Polyelectrolyte Multilayers Improve Osteogenic Differentiation of Adipose-Derived Stem Cells on Titania Nanotube Surfaces. Carbohydr. Polym..

[29] Raafat D., Sahl H.-G. (2009). Chitosan and Its Antimicrobial Potential—A Critical Literature Survey. Microb. Biotechnol..

[30] Rimoli C.V., de Oliveira Pedro R., Miranda P.B. (2022). Interaction Mechanism of Chitosan Oligomers in Pure Water with Cell Membrane Models Studied by SFG Vibrational Spectroscopy. Colloids Surf. B Biointerfaces.

[31] Nandi S., Guha P. (2018). Modelling the Effect of Guar Gum on Physical, Optical, Barrier and Mechanical Properties of Potato Starch Based Composite Film. Carbohydr. Polym..

[32] Kolya H., Kang C.-W. (2021). Polyvinyl Acetate/Reduced Graphene Oxide-Poly (Diallyl Dimethylammonium Chloride) Composite Coated Wood Surface Reveals Improved Hydrophobicity. Prog. Org. Coat..

[33] Inhibition Effects of Dilute-Acid Prehydrolysate of Corn Stover on Enzymatic Hydrolysis of Solka Floc|SpringerLink. https://link.springer.com/article/10.1007/s12010-011-9355-3.

[34] León Peláez A.M., Serna Cataño C.A., Quintero Yepes E.A., Gamba Villarroel R.R., De Antoni G.L., Giannuzzi L. (2012). Inhibitory Activity of Lactic and Acetic Acid on Aspergillus Flavus Growth for Food Preservation. Food Control.

[35] López M.J., Nichols N.N., Dien B.S., Moreno J., Bothast R.J. (2004). Isolation of Microorganisms for Biological Detoxification of Lignocellulosic Hydrolysates. Appl. Microbiol. Biotechnol..

[36] Kannisto M.S., Mangayil R.K., Shrivastava-Bhattacharya A., Pletschke B.I., Karp M.T., Santala V.P. (2015). Metabolic Engineering of *Acinetobacter baylyi* ADP1 for Removal of *Clostridium butyricum* Growth Inhibitors Produced from Lignocellulosic Hydrolysates. Biotechnol. Biofuels.

[37] Tsai C., Mitton K.P., Johnson B.F. (1989). Acetate Assimilation by the Fission Yeast, Schizosaccharomyces Pombe. Biochem. Cell Biol..

[38] Schneider H. (1996). Selective Removal of Acetic Acid from Hardwood-Spent Sulfite Liquor Using a Mutant Yeast. Enzym. Microb. Technol..

[39] Saadat S., Rawtani D., Braganza V. (2022). Antimicrobial Activity of Chitosan Film Containing Nanocomposite of *Trachyspermum ammi* (Ajwain) Seed Oil Loaded Halloysite Nanotubes against Foodborne Pathogenic Microorganisms. Appl. Clay Sci..

[40] Martins A.F., Facchi S.P., Follmann H.D.M., Pereira A.G.B., Rubira A.F., Muniz E.C. (2014). Antimicrobial Activity of Chitosan Derivatives Containing *N*-Quaternized Moieties in Its Backbone: A Review. Int. J. Mol. Sci..

[41] Fajardo A., Pereira A., Martins A., Paulino A., Muniz E., Hsieh Y.-L. (2016). Chitin and Chitosan-Based (NANO) Composites. Handbook of Composites from Renewable Materials.

[42] Machado B.R., Roberto S.B., Bonafé E.G., Camargo S.E.A., Camargo C.H.R., Popat K.C., Kipper M.J., Martins A.F. (2019). Chitosan Imparts Better Biological Properties for Poly(ε-Caprolactone) Electrospun Membranes than Dexamethasone. J. Braz. Chem. Soc..

[43] Arslan B., Soyer A. (2018). Effects of Chitosan as a Surface Fungus Inhibitor on Microbiological, Physicochemical, Oxidative and Sensory Characteristics of Dry Fermented Sausages. Meat Sci..

[44] Xing K., Zhu X., Peng X., Qin S. (2015). Chitosan Antimicrobial and Eliciting Properties for Pest Control in Agriculture: A Review. Agron. Sustain. Dev..

[45] Barbosa H.F.G., Francisco D.S., Ferreira A.P.G., Cavalheiro É.T.G. (2019). A New Look towards the Thermal Decomposition of Chitins and Chitosans with Different Degrees of Deacetylation by Coupled TG-FTIR. Carbohydr. Polym..

[46] Raj V., Kim Y., Kim Y.-G., Lee J.-H., Lee J. (2022). Chitosan-Gum Arabic Embedded Alizarin Nanocarriers Inhibit Biofilm Formation of Multispecies Microorganisms. Carbohydr. Polym..

[47] Costa E.M., Silva S., Veiga M., Vicente S., Tavaria F.K., Pintado M.E. (2017). Investigation of Chitosan’s Antibacterial Activity against Vancomycin Resistant Microorganisms and Their Biofilms. Carbohydr. Polym..

[48] Mecanismos de Resistência de Candida albicans aos Antifúngicos Anfotericina B, Fluconazol e Caspofungina. https://www.rbac.org.br/artigos/mecanismos-de-resistencia-de-candida-albicans-aos-antifungicos-anfotericina-b-fluconazol-e-caspofungina/.

[49] Rodríguez A.B.F., Menéndez D.C., Delgado E.O., Díaz O.L., Pino J.C.C. (2007). Evaluation of Chitosan as an Inhibitor of Soil-Borne Pathogens and as an Elicitor of Defence Markers and Resistance in Tobacco Plants. Span. J. Agric. Res..

[50] Elagamey E., Abdellatef M.A.E., Arafat M.D.Y. (2022). Proteomic Insights of Chitosan Mediated Inhibition of *Fusarium oxysporum* f. Sp. Cucumerinum. J. Proteom..

[51] Ren J., Tong J., Li P., Huang X., Dong P., Ren M. (2021). Chitosan Is an Effective Inhibitor against Potato Dry Rot Caused by *Fusarium oxysporum*. Physiol. Mol. Plant Pathol..

[52] Alburquenque C., Bucarey S.A., Neira-Carrillo A., Urzúa B., Hermosilla G., Tapia C.V. (2010). Antifungal Activity of Low Molecular Weight Chitosan against Clinical Isolates of *Candida* spp.. Med. Mycol..

[53] Younes I., Rinaudo M. (2015). Chitin and chitosan preparation from marine sources. Structure, properties and applications. Mar. Drugs.

[54] Garcia L.G.S., De Melo Guedes G.M., Fonseca X.M.Q.C., Pereira-Neto W.A., Castelo-Branco D.S.C.M., Sidrim J.J.C., De Aguiar Cordeiro R., Rocha M.F.G., Vieira R.S., Brilhante R.S.N. (2020). Antifungal Activity of Different Molecular Weight Chitosans against Planktonic Cells and Biofilm of Sporothrix Brasiliensis. Int. J. Biol. Macromol..

[55] Verlee A., Mincke S., Stevens C.V. (2017). Recent Developments in Antibacterial and Antifungal Chitosan and Its Derivatives. Carbohydr. Polym..

[56] Dhawale P.V., Vineeth S.K., Gadhave R.V., Fatima M.J.J., Supekar M.V., Thakur V.K., Raghavan P. (2022). Tannin as a Renewable Raw Material for Adhesive Applications: A Review. Mater. Adv..

[57] Suriyaprom S., Mosoni P., Leroy S., Kaewkod T., Desvaux M., Tragoolpua Y. (2022). Antioxidants of Fruit Extracts as Antimicrobial Agents against Pathogenic Bacteria. Antioxidants.

[58] Mila I., Scalbert A., Expert D. (1996). Iron Withholding by Plant Polyphenols and Resistance to Pathogens and Rots. Phytochemistry.

[59] Ucella-Filho J.G.M., da Freire A.S.M., Carréra J.C., Lucas F.M.F., Zucolotto S.M., Dias Júnior A.F., Mori F.A. (2022). Tannin-Rich Bark Extract of Plants as a Source of Antimicrobial Bioactive Compounds: A Bibliometric Analysis. S. Afr. J. Bot..

[60] Wrońska N., Katir N., Nowak-Lange M., El Kadib A., Lisowska K. (2023). Biodegradable Chitosan-Based Films as an Alternative to Plastic Packaging. Foods.

[61] Malerba M., Cerana R. (2018). Recent Advances of Chitosan Applications in Plants. Polymers.

[62] Münstermann N., Weichold O. (2024). Chitosan Itaconate Based Water- and Stain-Repellent Coatings for Wood. Prog. Org. Coat..

[63] Oliveira J.T.D., Tomasello M., Silva J.D.C. (2005). Resistência Natural Da Madeira de Sete Espécies de Eucalipto Ao Apodrecimento. Rev. Árvore.

[64] Facchi S.P., Souza P.R., De Almeida D.A., Madruga L.Y.C., Rosseto P., De Carvalho Nunes W.M., Kipper M.J., Martins A.F., Cardozo-Filho L. (2023). Surface Coatings Based on Chitosan and Tannins Applied in the in Vivo Prevention of Corn Streak Disease. Chem. Eng. J..

[65] Sacco F.S., Cardozo-Filho L., Massa T.B., Feihrmann A.C., Abrantes K.K.B. (2020). Unidade de Ultra Pressurização para Impregnação e Esterelização de Amostras em Meio Liquído. Patent.

[66] Macías-Almazán A., Lois-Correa J.A., Domínguez-Crespo M.A., López-Oyama A.B., Torres-Huerta A.M., Brachetti-Sibaja S.B., Rodríguez-Salazar A.E. (2020). Dataset of Operating Conditions to Isolate Cellulose Nanocrystalline from Sugarcane Bagasse and Pinewood Sawdust as Possible Material to Fabricate Polymer Electrolyte Membranes. Data Brief.

[67] De Oliveira A.C., Madruga L.Y.C., Chevallier P., Copes F., Mantovani D., Vilsinski B.H., Popat K.C., Kipper M.J., Souza P.R., Martins A.F. (2024). Polyphenolic Tannin-Based Polyelectrolyte Multilayers on Poly(Vinyl Chloride) for Biocompatible and Antiadhesive Coatings with Antimicrobial Properties. Prog. Org. Coat..

[68] Souza P.R., Vilsinski B.H., de Oliveira A.C., Berton S.B.R., Madruga L.Y.C., Schrekker H.S., Radovanovic E., Kipper M.J., Martins A.F., Muniz E.C. (2022). Poly(Ethylene Terephthalate) Films Coated with Antimicrobial Gelatin/Chondroitin Sulfate Polyelectrolyte Multilayers Containing Ionic Liquids. Prog. Org. Coat..

[69] Facchi S.P., de Oliveira A.C., Bezerra E.O.T., Vlcek J., Hedayati M., Reynolds M.M., Kipper M.J., Martins A.F. (2020). Polycationic Condensed Tannin/Polysaccharide-Based Polyelectrolyte Multilayers Prevent Microbial Adhesion and Proliferation. Eur. Polym. J..

[70] da Silva Bruni A.R., de Souza Alves Friedrichsen J., de Jesus G.A.M., da Silva Alves E., da Costa J.C.M., Souza P.R., de Oliveira Santos Junior O., Bonafe E.G. (2023). Characterization and Application of Active Films Based on Commercial Polysaccharides Incorporating ZnONPs. Int. J. Biol. Macromol..

[71] (2022). Laboratory Method for Evaluating the Decay Resistance of Wood-Based Materials against Pure Basidiomycete Cultures: Soil/Block Test.

