# Supplementary material for: Ultra-Pressurized Deposition of Hydrophobic Chitosan Surface Coating on Wood for Fungal Resistance
Source: Int J Mol Sci. 2024 Oct 10;25(20):10899. doi: 10.3390/ijms252010899 (PMC11507266; doi:10.3390/ijms252010899)
Supplement: Supplementary file 1 [file ijms-25-10899-s001.zip › ijms-3245076-supplementary.pdf]

Supplementary material

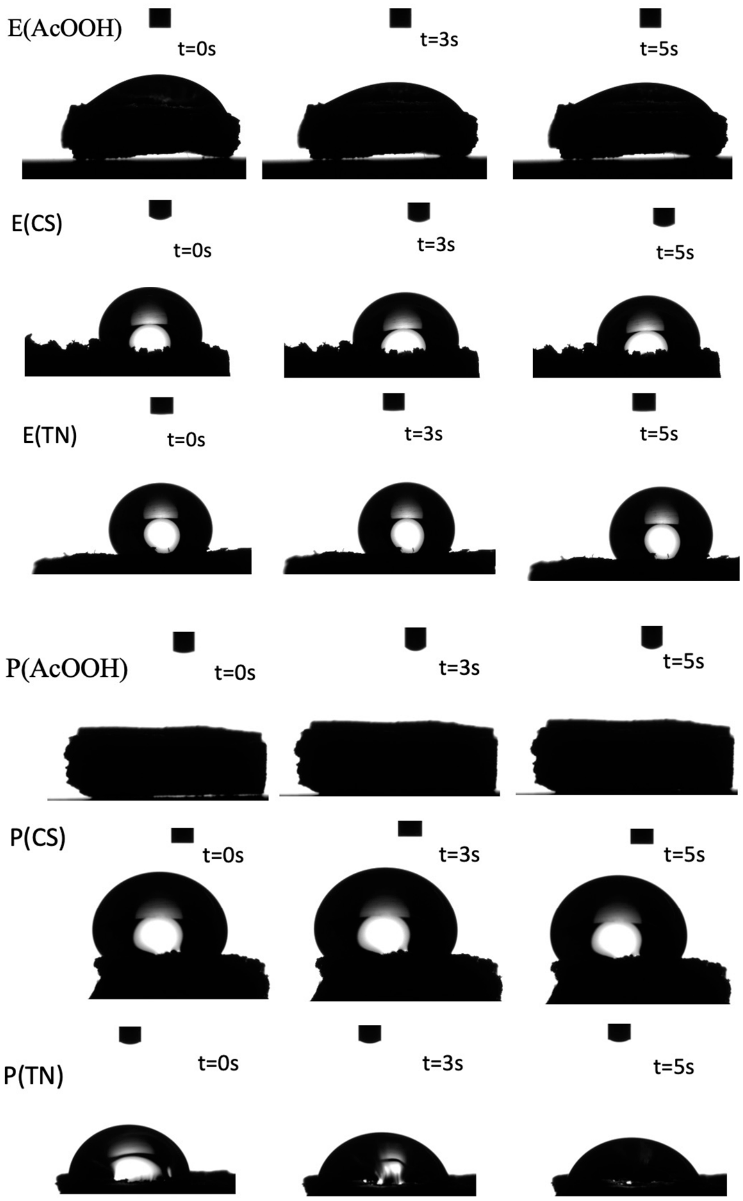

Figure S1. Digital images of the water droplets deposited on the *Pinus* sp (P). and *Eucalyptus* sp (E) samples for water contact angle (WCA) measurements. Codes: P(AcOOH) is the *Pinus* sp. coated AcOOH, P(TN) is the *Pinus* sp. coated with TN, P(CS) is the *Pinus* sp. coated with CS, E(AcOOH) is the *Eucalyptus* sp. coated with AcOOH, E(TN) is the *Eucalyptus* sp. coated with TN, and E(CS) is the *Eucalyptus* sp. coated with CS.

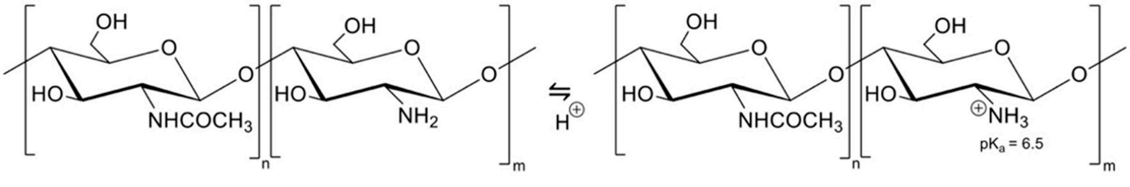

Figure S2. Chemical structures of the partially ionized CS chains.

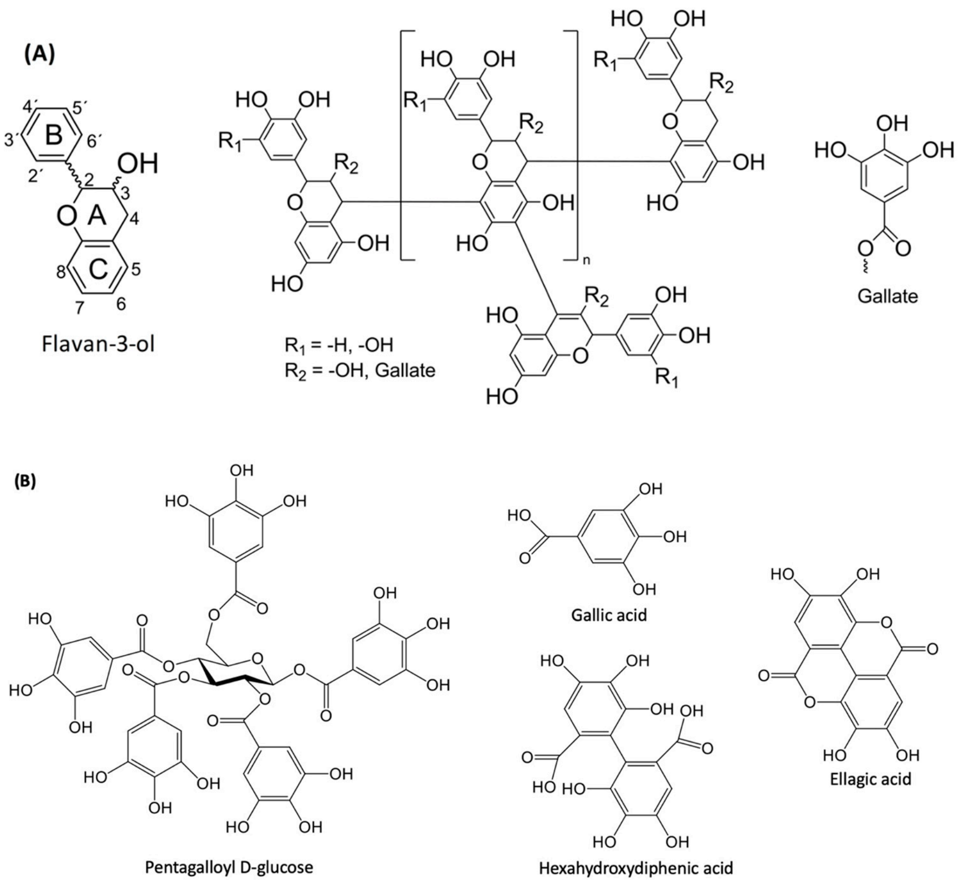

Figure S3. Chemical structures of condensed tannins (**A**) and hydrolyzable tannins (**B**). The structure of TN comprises both condensed and hydrolyzable tannins.

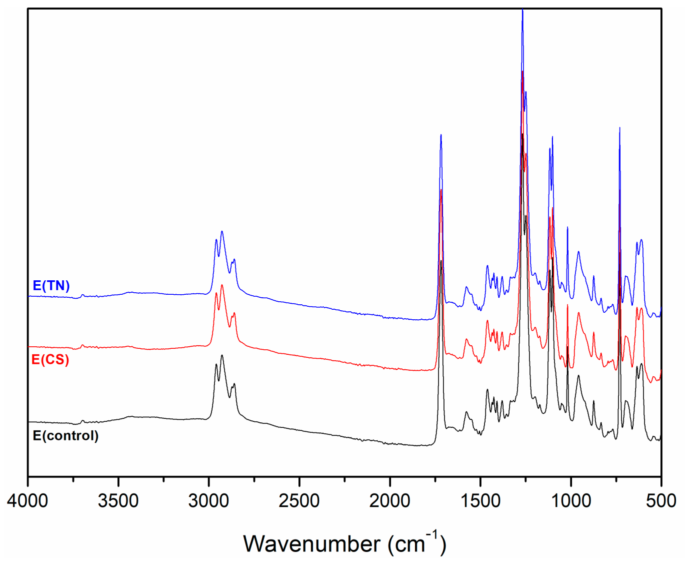

Figure S4. FTIR-ATR spectra of *Eucalyptus* sp. samples coated with tannin (E(TN)) and chitosan (E(CS)), as well as the uncoated *Eucalyptus* sp. Sample (E(control).

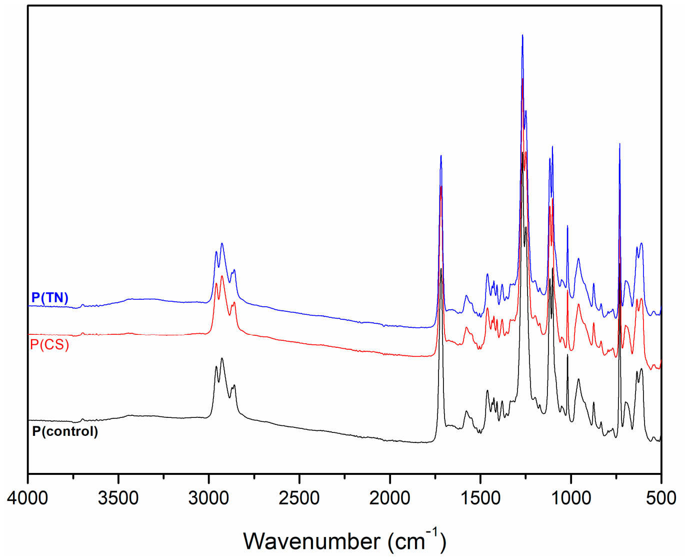

Figure S5. FTIR-ATR spectra of *Pinus* sp. samples coated with tannin (P(TN)) and chitosan (P(CS)), as well as the uncoated *Pinus* sp. Sample (P(control)).

Chitosan and Tannin Characterization

Figure S6 shows the FTIR-ATR spectra of CS and TN. The CS FTIR-ATR spectrum exhibits characteristic bands associated with the CS structure. The bands at 1642 and 1592 cm^{-1} are ascribed to the C=O stretching of amide and angular deformation of N–H, respectively. The bands in the range of 1000 to 1150 cm^{-1} are assigned to the oxygenated groups on CS (C–O, C–OH, and C–O–C), comprising ether and primary alcohol functional groups. The band at 1373 cm^{-1} is attributed to C–H bonds on acetylated moieties. The other signals in the CS FTIR-ATR spectrum are associated with the polymer structure (Figure S2). Specifically, the bands at 3355 and 3285 cm^{-1} are attributed to –OH and N–H bonds, respectively [32,33].

The TN FTIR-ATR spectrum exhibits a band at 1730 cm^{-1} attributed to the C=O sites in hydrolyzable tannins (Figure S3). The signals at 1603 and 1450 cm^{-1} are related to the C=C stretching in the aromatic rings of condensed tannins (Figure S3). The band at 1024 cm^{-1} is associated with C–O in phenol, ether, and alcohol functional groups. The bands at 1317 and 3228 cm^{-1} are also assigned to the C–O and –OH bonds [34,35].

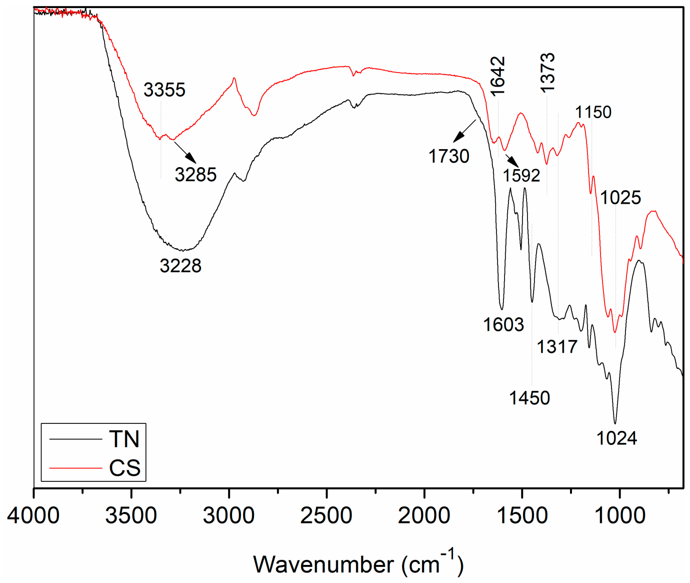

Figure S6. FTIR-ATR spectra of the CS and TN.

The Zeta potential and hydrodynamic radius of CS and TN aqueous solutions at 0.5 mg/mL were determined through DLS measurements. CS solution was prepared in a 1.0% *v*/*v* diluted AcOOH acid solution, while the TN solution was prepared in distilled water. Despite CS's insolubility in water, it exhibits solubility in weak and diluted organic acid solutions. The AcOOH aqueous solution induces partial ionization of CS [36], leading to partially protonated

polymer chains (Figure S2). Protonated CS interacts with water molecules via ion-dipole forces established between the protonated $-NH_3^+$ groups (pK_a 6.5) and water molecules, facilitating CS solubilization [37]. The $-NH_3^+$ groups and partial CS ionization in a diluted AcOOH solution contribute to a Zeta potential of +18.8 mV. The pH of the CS solution (0.5 mg/mL) in AcOOH is 4.2 [38]. The hydrodynamic radius of the CS in solution is 445 nm.

Figure S3 (Supplementary Material) presents the chemical structures of the primary constituents of TN. Tannins interact with proteins and polysaccharides, forming stable complexes [39]. The chemical structure of condensed tannins (polyphenols) is shown in Figure S3A, whereas Figure S3B exhibits the primary hydrolyzable tannin chemical structures [40]. Condensed tannins are characterized by flavonoid units (flavan-3-ol) linked through C-C bonds between the carbon atoms C_4 and C_8 in the catechin or flavan-3-ol unit (Figure S3a). This C-C bond is termed the "condensed bond", a feature associated with the condensed tannins [41]. Hydrolyzable tannins mainly comprise glucose units esterified with gallic or ellagic acids (Figure S3B) [41,42]. These tannins can undergo hydrolysis through either chemical or enzymatic processes.

The TN solution has a negative Zeta potential of −23.1 mV. Tannins are rich in phenolic groups with a pK_a close to 7.0 and carboxylic acids with a pK_a between 4.0 and 4.5 (Figure S3). These groups on TN support the negative Zeta potential of −23.1 mV [43–46]. The hydrodynamic radius of TN in an aqueous solution is 615 nm. Dimers of gallic acid (hexahydroxidiphenic acid), gallic acid, and ellagic acid may occur in TN solutions, contributing to the negative Zeta potential, as these substances undergo partial ionization in aqueous media (Figure S3B).
